# Mesotherapy with HA and Choline Against Facial Skin Aging: An Open-Label Uncontrolled, Monocentric Study

**DOI:** 10.3390/jcm14072303

**Published:** 2025-03-27

**Authors:** Antonio Scarano, Erda Qorri, Andrea Sbarbati, Vincenzo Desiderio, Domenico Amuso

**Affiliations:** 1 Department of Innovative Technology in Medicine and Dentistry, University of Chieti-Pescara, 66100 Chieti, Italy; 2 Department of Dentistry, Faculty of Medical Sciences, Albanian University, 1001 Tirana, Albania; erda79@yahoo.com; 3 Department of Neurosciences, Biomedicine and Movement Sciences, Anatomy and Histology Section, School of Medicine, University of Verona, 81100 Verona, Italy; andrea.sbarbati@univr.it (A.S.); d.amuso.estetica@gmail.com (D.A.); 4 Department of Experimental Medicine, University of Campania “Luigi Vanvitelli” Via L. Armanni 5, 80138 Napoli, Italy; vincenzo.desiderio@unicampania.it

**Keywords:** hyaluronic acid, choline, face rejuvenation, face revitalization, skin aging

## Abstract

**Background**: Facial aging involves soft and hard tissues with changes that can affect an individual’s self-esteem and aesthetic appearance. Techniques used to counteract these changes include the use of solutions to be injected into the dermis, such as dermal matrix, vitamins, and antioxidants. B vitamins and choline are vital nutrition for humans and many other animals (vitamin B4), required to produce acetylcholine (ACh). It is considered a neurotransmitter universal methyl donator and of the major membrane constituent phosphatidylcholine (PC) and is crucial for the functioning of cell membranes, including those in skeletal muscle cells. The aim of this study is to evaluate the efficacy and safety of a fragment of HA amino acid and choline in a solution of phosphate buffer system used via mesotherapy. Specifically, state that the primary endpoint was the efficacy assessment using the Scientific Assessment Scale of Skin Quality (SASSQ), while secondary endpoints included safety assessments and patient-reported outcomes. **Methods:** Forty (40) subjects completed the study. In total, 40 subjects were screened and included in the study. The total duration of the study was 14 months. The first subject was included on 12 January 2019, and the last subject’s last visit was on 14 March 2020. All patients received the SKIN Colin^®^ products by mesotherapy technique for 8 weeks, providing the treatment with the use of 0.5 cc syringes and 13 mm long, 30 G diameter needles. The solution was inoculated into the deep layer of the dermis of the face with a suitable amount of at least 0.2/0.3 mL in the cutaneous points four times every 15 days. Each subject had to be followed for 168 days after the last mesotherapy session. Only enrolled subjects received the HA and choline via mesotherapy. The primary efficacy endpoint was the absolute change in the Scientific Assessment Scale of Skin Quality from Baseline (Day 0) to Day 168. A reduction of at least one point in the SASSQ was considered to reach the endpoint goal. **Results:** The results of the present investigation show Scientific Assessment Scale of Skin Quality (SASSQ) mean at baseline was 2463 with a standard deviation of 0.36, while at day 168, the mean was 1303 with a standard deviation of 0.36. The difference was statistically significant (*p* < 0.0001). Also, the GAIS was improved after treatment with Skin Colin^®^. The assessment of “satisfaction with treatment” was very high by the majority of subjects. **Conclusions:** In conclusion, our results suggest that a course of treatment with choline via mesotherapy results in an improvement of the Scientific Assessment Scale of Skin Quality. This data is very important for possible fields of application in the treatment of skin and muscle aging. However, the present study has limitations due to the small sample size and the lack of a control group.

## 1. Introduction

Facial aging involves soft and hard tissues with changes that can affect an individual’s self-esteem and aesthetic appearance.

Exogenous or extrinsic variables (chronic light exposure, pollution, ionizing radiation, chemicals, toxins) and endogenous or intrinsic factors (genetics, cellular metabolism, hormones, and metabolic processes) combine to influence the complicated biological process of skin aging [1]. Together, these elements cause cumulative physiological and structural changes, gradual variations in each layer of the skin, and changes in the look of the skin, particularly in parts of the skin exposed to the sun [2,3,4,5,6]. In photoaging, a gradual loss of skin elasticity is observed, leading to sagging. Prematurely photoaged skin typically presents with a thickened epidermis, patchy discoloration, deep wrinkles, laxity, dullness, and roughness [7,8,9,10,11]. This condition is accompanied by a reduction in epidermal turnover, resulting in decreased wound healing capacity and less effective desquamation, especially in the elderly. In contrast, aging due to intrinsic factors is characterized by wrinkled, thin, and dehydrated skin [12]. Techniques used to counteract these changes include the use of sunscreen creams, peelings, fillers, and mesotherapy [13,14,15].

Researchers propose various solutions to be injected into the dermis, such as dermal matrix, vitamins, and antioxidants. SKIN-COLIN^®^ is a filling and modeling solution for the dermal matrix based on hyaluronic acid with choline for restoring the colloidal layer of the dermis. Formerly categorized as a B vitamin, choline is vital nutrition for humans and many other animals (vitamin B4) is required to produce acetylcholine (ACh). It is considered a neurotransmitter universal methyl donator and of the major membrane constituent phosphatidylcholine (PC) and is crucial for the functioning of cell membranes, including those in skeletal muscle cells [16]. Choline is used to replace the methyl groups of methionine and S-adenosylmethionine after being metabolized in the liver to betaine [17]. The interactions between phospholipids (PL) and hyaluronic acid (HA) are important for skin aging, lubrication of synovial joints, and vitreous humor. Acetylcholine influences the skin aging process at the cellular level through several mechanisms, such as modulating calcium influx into keratinocytes that decrease with aging and chronic photo exposure, suggesting a role in skin aging and potential as a marker of degenerative changes in the epidermis [18]. Acetylcholine influences the skin aging process at the cellular level through several mechanisms, such as modulating calcium influx into keratinocytes that decrease with aging and chronic photo exposure, suggesting a role in skin aging and potential as a marker of degenerative changes in the epidermis [18]).

Furthermore, acetylcholine and its receptors are involved in pathways related to skin aging, including epidermal barrier repair, collagen and elastin production, and inhibition of reactive oxygen species (ROS). These pathways are crucial for the maintenance of skin structure and function, and their modulation by acetylcholine affects the aging process [19].

For these reasons, hyaluronic acid and choline were associated with therapies for dry eye disease, synovial joint lubrication, and skincare products [20].

The intradermal solution SKIN Colin^®^, based on bio-fermentative hyaluronic acid HA fragment and choline, is indicated for the correction of skin damage, including rhytidosis, photoaging, and skin dehydration.

The current clinical investigation was designed to obtain further clinical safety and efficacy data for the SKIN Colin^®^ medical devices in the treatment of skin damage. Specifically, state that the primary endpoint was the efficacy assessment using the Scientific Assessment Scale of Skin Quality (SASSQ), while secondary endpoints included safety assessments and patient-reported outcomes. An open, uncontrolled study design was considered appropriate to evaluate the impact of the hyaluronic acid and choline solution on facial skin. Improvements were assessed with the Scientific Assessment Scale of Skin Quality (SASQ) [21]. As no spontaneous improvement of skin was expected, a comparison versus Baseline with an untreated control group was not considered necessary in this post-marketing setting. This decision was based on the understanding that the natural progression of skin aging would not result in significant changes without intervention.

The aim of this study is to evaluation of efficacy and safety of a fragment of HA amino acid and choline in a solution of phosphate buffer system used via mesotherapy. Specifically, state that the primary endpoint was the efficacy assessment using the Scientific Assessment Scale of Skin Quality (SASSQ), while secondary endpoints included safety assessments and patient-reported outcomes

## 2. Material and Methods

This clinical investigation was conducted under the auspices of the ethical principles originating in the Declaration of Helsinki and the International Standard ISO. The present study followed the STROBE guidelines for cross-sectional studies [22]. The clinical investigation was conducted took place at the Department of Medical Sciences of the University of Tirana, Albania, registered with Nr.374 Prot. Date 27 June 2019.

Before entry into the clinical investigation, the investigator had to explain to each subject the aims, methods, anticipated benefits, and potential hazards of the investigation. After these explanations and before entering the investigation, the subject had to voluntarily sign the informed consent statement and also receive a copy of the signed and dated form.

It adhered rigorously to ethical principles, including compliance with the World Medical Association Declaration of Helsinki (https://www.wma.net/wp-content/uploads/2018/07/DoH-Oct2008.pdf) (Accessed in the 2 January 2019) and the additional requirements stipulated by Albania law. SKIN-Colin^®^, an injectable medical device based on hyaluronic acid, amino acids, and choline intended for intradermal administration, was used in the present study. Skin Colin^®^ consists of linear HA obtained from Streptococcus zooepidemicus, formulated to a concentration of 0.1% in a physiological buffer. It belongs to risk class III in accordance with Directive 93/42/EEC (Annex IX, Regulation 8) and Regulation (EU) 2017/745 (Annex VIII, Regulation 8) [23]. HA is mainly produced through bacterial fermentation with Streptococcus zooepidemicus. This method has become more widespread due to changes in legislation and the efficiency of microbial fermentation. It is also widely used in the commercial production of HA due to its short fermentation cycle and high production intensity, making it suitable for large-scale industrial applications. SKIN-Colin^®^ is a firming and shaping solution of the dermal indicated in the treatment of skin damage to the face. Active ingredients are Sodium hyaluronate 0.10%, Choline Bitartrate 0.15%, L-lysine hydrochloride 0.18%, L-proline 0.20%, glycine 0.20%, L-serine 0.20%, L-alanine 0.24%, L-cysteine 0.005%, Sodium Chloride 0.21%, Bibasic sodium phosphate dihydrate 0.95%, Monobasic sodium phosphate dihydrate 0.22%, Water ppi Qb to 100. SKIN Colin^®^ is intended to be used in adults (at least 18 years old) to treat skin damage. The device is for single use, sterile, non-active, and without measuring function. The investigational device had to be stored below 25 °C and protected from light, heat, and frost [24]. The objectives of the investigation were to evaluate the efficacy and safety of SKIN Colin^®^ in the treatment of skin damage. The efficacy of the treatments was assessed by measuring the Scientific Assessment Scale of Skin Quality [21] (SASSQ) before the treatments and right after the last session of the treatment protocol (day 168). The primary endpoint was considered met with an improvement of at least 1 point in the SASSQ.

The criteria for inclusion were: male or female subjects between 30 years and 65 years, presence of rhytidids, dehydration pigmentation affecting the face having a total mean score ≥2 in the Scientific Assessment Scale of Skin Quality, healthy skin free of diseases that could interfere in cutaneous aging evaluation, willingness to abstain from any cosmetic or surgical procedures in the treatment area for the duration of the clinical investigation, willingness to refrain from any surgical operations, including the use of botulinum toxin, throughout the length of the clinical investigation, willingness to refrain from gaining or losing more than 10% of one’s body weight during the course of the clinical trial, written informed consent. Patients with the following were excluded: pregnancy, lactating, planned pregnancy or unwilling to use contraceptives, history of mental disorders or emotional instability, history of allergic reaction to HA products, skin of the to-treat region affected by cosmetic treatments (e.g., laser therapy within the last 12 months, chemical peeling within the last 3 months, dermabrasion within the last 12 months, botulinum toxin within the last 12 months), connective tissue diseases, diabetes mellitus or uncontrolled systemic diseases, immune deficiency virus-positive individuals, presence of silicone implant or another non-absorbable substance (permanent fillers) in the area of product application, cutaneous lesions in the evaluated area, previous (within 30 days of enrollment) treatment with another investigational drug and/or medical device or participation in another clinical study.

All enrolled patients were to receive eight injections of the investigational device into the face in regions where correction was needed. The injection volume had to be estimated by the investigator and recorded in the CRF. All patients received the SKIN Colin^®^ products by mesotherapy technique, providing the treatment with the use of 0.5 cc syringes with 13 mm long, 30 G diameter needles. The solution was inoculated into the deep layer of the dermis of the face with a suitable amount of at least 0.2/0.3 mL in the cutaneous points four times every 15 days.

Each subject had to be followed 168 days after the last mesotherapy session.

All treatments given in addition to the investigational device and all previous treatments given within 7 days before Day 0 of the investigation were to be recorded in the CRF, together with the indication, quantity, or dose administered, and dates and time of administration. Subjects were to be questioned concerning any new medications or changes in current medications. In particular, the use of anticoagulation or antiplatelet agents (e.g., aspirin) had to be identified.

The following medications were prohibited: two days before Day 0 and before each injection: acetylsalicylic acid, vitamin E, Gingko Biloba, vasodilators, amino salicylic acid, non-steroidal anti-inflammatory drugs, platelet aggregation inhibitors, topical and systemic retinoids, immunosuppressive medication for the entire duration of the clinical investigation.

After written informed consent was obtained, the following assessments were planned: evaluation of inclusion and exclusion criteria.

Demography, medical history (including information on prior facial cosmetic or surgical procedures), prior medication, urine pregnancy test, face photograph, weight measurement, and SASSQ assessment.

Subjects who complied with in and exclusion criteria underwent SKIN Colin^®^ application and further assessments were scheduled as listed below: concomitant medication, AE collection.

### 2.1. Scientific Assessment Scale of Skin Quality

The Scientific Assessment Scale of Skin Quality [21] represents an innovative, universal, and reliable measurement instrument for a valid and reproducible evaluation of seven parameters of aged skin quality, as described by Christine Eiben-Nielson et al. [21]. Each parameter is evaluated on a 0–4 intensity scale (0 = no damage, 4 = very severe damage)

GAIS is a 5-point scale that rates global aesthetic improvement in appearance compared to pre-treatment, as judged by the investigator. The rating categories were “worse = 0”, “no change = 1”, “improved = 2”, “much improved = 3”, and “very much improved = 4”.

To assess the subject’s satisfaction, subjects were asked to indicate their level of satisfaction on 3- or 5-point scales as follows:

• Question 1: How would you judge the change in your appearance after treatment. Very much improved, much improved, slightly improved, no change, worsened.
• Question 2: How satisfied are you with the treatment, very satisfied, satisfied, not satisfied.

### 2.2. Statistical and Determination of Sample Size

Based on the literature, 80% of subjects reached an improvement of at least 1 SASSQ point by Day 120. Using a mean difference of 1 on SASSQ, a standard deviation of 10%, a power of 80%, and a 2-sided significance level of 5%, 38 subjects were needed. This sample size was considered sufficient to adequately investigate the objectives of the investigation.

Statistical analyses were conducted using GraphPad Prism 10. Continuous variables were summarized as mean ± standard deviation (SD). The primary endpoint, the change in the Scientific Assessment Scale of Skin Quality (SASSQ) from baseline to Day 168, was analyzed using a Wilcoxon signed-rank test. A *p*-value < 0.05 was considered statistically significant. There were no missing data or dropouts, as all 40 subjects completed the study.

## 3. Results

**Figure 1:**
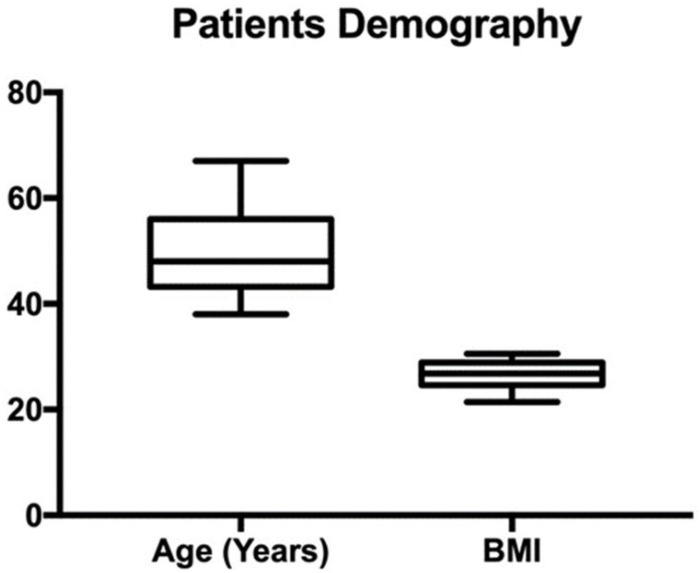
Patient’s demography (age, BMI).

The total duration of the study was 14 months. The first subject was included on 12 January 2019, and the last subject’s last visit was on 14 March 2020. Only enrolled subjects received the investigation device. In total, forty subjects were selected and included in the study. All 40 patients completed the study. All patients were treated at least once with the device under study and were included in the SAF. None of the patients had protocol deviations. Subject demography at Baseline is summarized in Table 1 and Figure 1. All participants in the study were female (100%). The age ranged from 38 to 67 years, with a median age of 48 years. The Body Mass Index (BMI) ranged from 21.4 to 30.6, with a median range of 26.8. All subjects were Caucasian, of Albanian and Italian descent. Most female subjects were of childbearing potential, and pregnancy test results were negative. Figure 1 and Table 1 provide an analysis of the demographic details.

**Figure 2:**
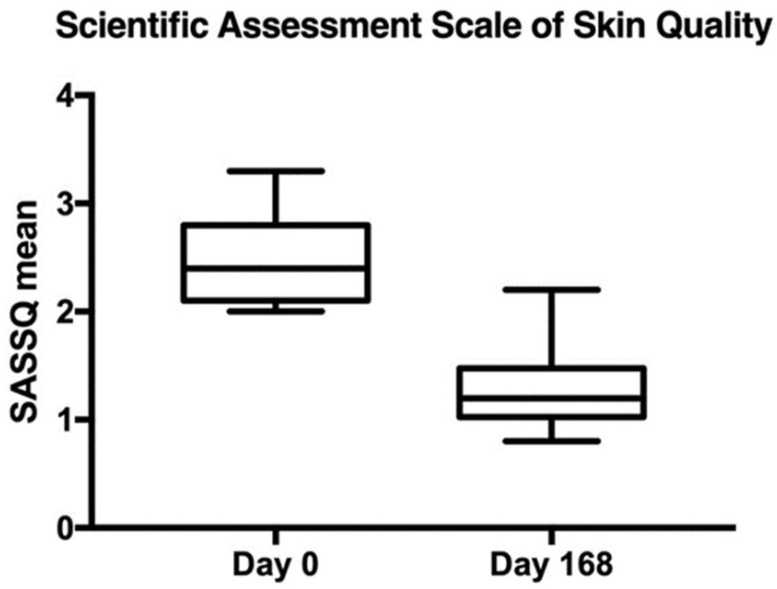
Difference in the SASSQ from day 0 to day 168 (N = 40). Error bars represent 95% confidence interval. *p* < 0.0001.

The primary efficacy endpoint was the absolute change in the Scientific Assessment Scale of Skin Quality from Baseline (Day 0) to Day 168. A reduction of at least one point in the SASSQ was considered to reach the endpoint goal. The SASSQ mean at baseline was 2463 with a standard deviation of 0.36, while on day 168, the mean was 1303 with a standard deviation of 0.36. The difference was statistically significant (*p* < 0.0001). Figure 2 visually represents this change, including confidence intervals to indicate variability. A reduction of at least one point in SASSQ is considered clinically meaningful, suggesting that the treatment led to a noticeable improvement in skin quality. The consistency in the standard deviation across both points further supports the reliability of these findings. The plotted data in Figure 2 visually reinforces this significant change.

Most subjects rated appearance and the treatment positive and would recommend it to others. The subjects’ judgment of appearance after treatment changed with time; however, by the termination of the study on Day 168, 37.5% of subjects still considered their appearance very much improved, and 42.5% still considered their appearance much improved. The assessment of “satisfaction with treatment” was very high by the majority of subjects (Table 2).

**Figure 3:**
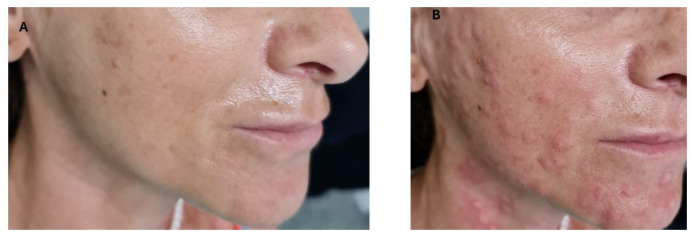
(**A**) Photo before the treatment. (**B**) immediately after the treatment.

Figure 3 highlights the appearance before and immediately after mesotherapy treatment.

### 3.1. Global Aesthetic Improvement Scale

The physician evaluated the appearance of the defect treated in accordance with GAIS at the final visit (day 168) compared to Day 0 as “very much improved” for 10 patients (22.5%), “much improved” for 16 patients (40%) and as “improved” for 14 patients (35,0%). None of the patients was judged as having “no change”. Injection site bruising, injection site pain, and injection site redness (reported by four, two, and two subjects, respectively) were the only AEs reported by more than one subject (Table 3). The AEs were mostly pain and redness, were short-lived, and did not require any treatment.

### 3.2. Histological Results

**Figure 4:**
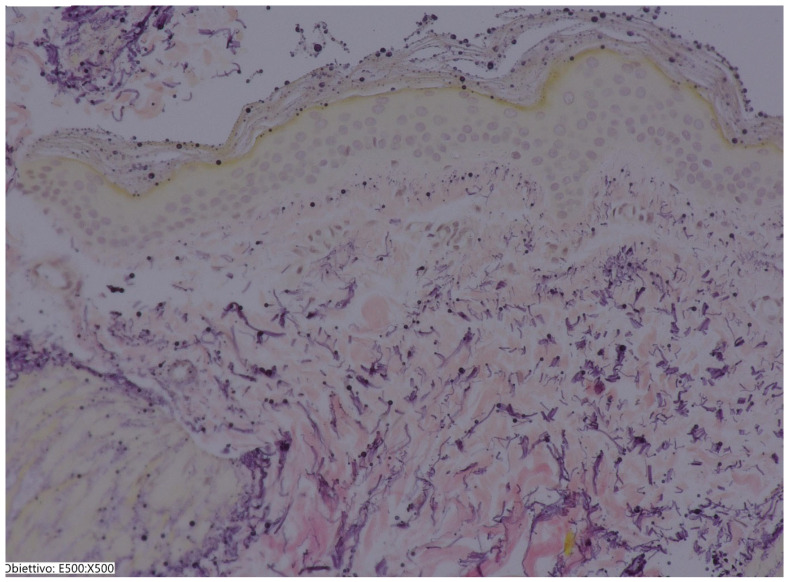
Sample before treatment. A few vessels and a thin epithelium are observed. Hematoxylin eosin staining 40×. (Sample before treatment. A few vessels and a thin epithelium are observed. Hematoxylin eosin staining 40×).

**Figure 5:**
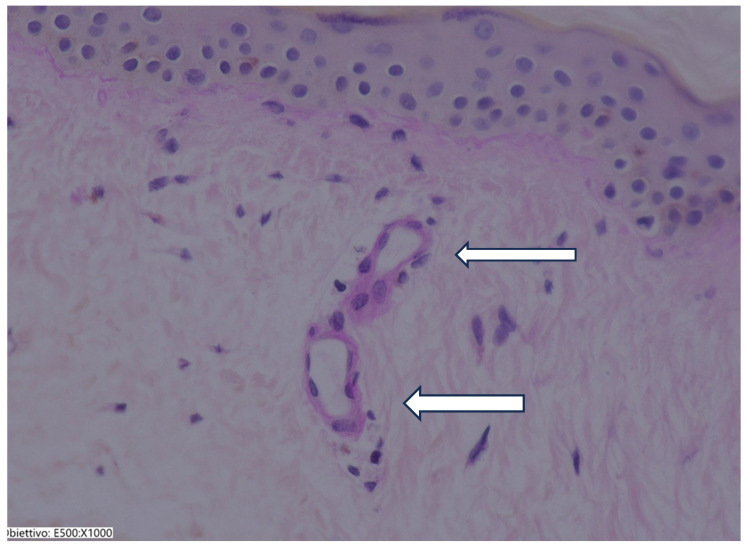
Sample after treatment. An increase in vessels (Arrows) and thickening of the epithelium is observed. Hematoxylin eosin staining 40×. (Sample after treatment. An increase in vessels and thickening of the epithelium is observed. Hematoxylin eosin staining 40×).

A biopsy of the patient’s skin was taken on day 0 and on day 168 to evaluate the effect of SKIN Colin^®^ on the connective tissue arrangement in the dermis. The biopsies were performed on the cheek or near the lip. Forty biopsies with a 1.0 mm diameter and 3 mm in length were sampled in all patients by means of a specific punch (Kai Medical, Oyana, Japan). For the histological evaluations, 10 fields were selected for each sample, each measuring 2000 × 2000 microns. These evaluations were conducted using a KEYENCE digital microscope (Osaka, Japan). The evaluation was performed by two independent pathologists and evidenced a significant improvement in dermis organization and in the structure of microvessels. An increase in the thickness dermis was observed. Many vessels were detected after treatment. This strongly points out the efficacy of SKIN Colin^®^ in supporting skin health and recovery from damage (Figure 4 and Figure 5). No acute inflammatory cells or tissue necrosis were observed.

## 4. Discussion

Mesotherapy is a form of alternative medicine that involves intradermal or subcutaneous injections of pharmaceutical preparations with 30 or 33 G needles. This technique is used in various fields of medicine to alleviate muscle pain, tendinopathies, and other painful conditions to treat local inflammation and cellulite.

The solution was inoculated into the deep layer of the dermis of the face with a suitable amount of at least 0.2/0.3 mL in the cutaneous points four times every 15 days. This study aimed to evaluate whether choline administration plays an adjuvant role in reducing skin aging. The results of this investigation show SASSQ mean at baseline was 2463 with a standard deviation of 0.36, while at day 168, the mean was 1303 with a standard deviation of 0.36. The difference was statistically significant (*p* < 0.0001). Also, the GAIS was improved after treatment with Skin Colin^®^. These enhancements in SASSQ and GAIS were corroborated histologically by improvements in dermal organization and an increase in the number of vessels. The presence of a major vessel likely played a role in influencing the regenerative and healing processes.

SKIN Colin^®^ is a biodegradable HA solution intended to be used in adults (at least 18 years old) for the treatment of skin damage.

Our study has elaborated on the beneficial effects of Ha fragment and Choline in protecting skin aging in humans, these effects include the improvement of SASSQ and GAIS. The histological findings show an improvement in dermis organization, an increase in vessel density, and the absence of inflammatory cells. This suggests that the treatment does not induce adverse inflammatory responses or tissue damage, which is crucial for its efficacy and patient safety. The histological outcome of the present study underscores the potential of SKIN Colin^®^ to support skin health and recovery from damage without causing harmful side effects.

Overall, these histological results strongly point to the efficacy of SKIN Colin^®^ in improving dermal structure and microvascular health, making it a promising option for skin rejuvenation.

In the present clinical investigation, the safety and efficacy of SKIN Colin^®^ were evaluated in the treatment of skin damage, as evidenced by the SASSQ assessment. Analysis of the results and the primary endpoint of effectiveness, measured by the absolute change in the Scientific Assessment Scale of Skin Quality from day 0 to day 168, showed significant improvement in all parameters analyzed, including an overall improvement in appearance and a reduction in wrinkles. The efficacy of SKIN Colin^®^ was further reflected in the positive rating by subjects (subject satisfaction) and investigators (GAIS) and from the histological analysis. Treatment with SKIN Colin^®^ was generally safe. Adverse effects included injection site redness, pain, and bruising, which were short-term adverse drug events (ADEs) already described in previous studies and are common for intradermal injections. Therefore, SKIN Colin^®^ is considered well-tolerated and can be a valuable tool for treating skin damage and counteracting the effects of aging. The treatment protocol lasted 168 days to allow for a comprehensive assessment of the efficacy of the treatments over a significant period. This duration ensures that any changes in the Scientific Assessment Scale of Skin Quality (SASSQ) are accurately measured and that the results are reliable and meaningful.

Approximately half of the hyaluronic acid (HA) in the human body is found in the skin [23], with the remaining portion being found in synovial fluid [24], the vitreous body [25], the umbilical cord [24,26], and areas including tendons, joints, pleura, sheaths, and the pericardium where friction is present. For these reasons, we have used a fragment of HA via mesotherapy with amino acid as a precursor of protein and choline for its role in skin pathophysiology. Choline is a precursor to phosphatidylcholine, a major component of cell membranes. This helps maintain the structure and function of skin cells. Choline contributes to the synthesis of acetylcholine, which is involved in muscle function and skin tone. This can help reduce the appearance of fine lines and wrinkles. It is hypothesized that elevated acetylcholine at neuromuscular synaptic junctions could be a key factor in enhancing facial muscle tensile strength and improving skin firmness. This suggests that while acetylcholine precursors may have some immediate skin-firming effects, their impact on long-term skin tightening and wrinkle reduction remains inconclusive and requires further research [27].

Non-neuronal acetylcholine is synthesized by human keratinocytes and acts as a local cell signaling molecule, regulating functions such as cell adhesion, proliferation, desmosomal cell contact (barrier function), and glandular activity [26,28]. In both the human central and peripheral nervous systems, acetylcholine functions as a neurotransmitter. Recent studies, however, show that the cholinergic system is widely expressed in human non-neuronal cells [29], such as bacteria epithelial cells (human airways, epidermis, alimentary tract) [26]. In fact, acetylcholine (ACh) is involved in cellular regulation, such as differentiation and cell proliferation [30,31]. Acetylcholine (Ach) appears to be required for the simultaneous activation of muscarinic and nicotinic receptors in order to balance and synchronize ionic and metabolic processes within the cell during epidermal renewal [32]. The Ach is a highly reactive molecule, and consequently, its use may lead to side effects; for these reasons, it is used in diluted form for certain skin diseases [28], such as poor surgery, acute illness, wound healing after chronic diseases or trauma. Acetylcholine (ACh) exerts its effects on keratinocytes through both nicotinic and muscarinic receptors. Choline is a vital micronutrient that plays a key part in several metabolic processes that support neurological, hematological, and hepatic homeostasis. A recent review emphasized the impact of choline on skeletal muscle, detailing its involvement in various pathways related to protein and fat metabolism, inflammation, and autophagy [33]. Many researchers have utilized diluted acetylcholine to achieve promising results for its role in promoting wound repair [27,34]. In the present study, we used choline as a precursor to acetylcholine. This approach is based on the understanding that choline, once administered via mesotherapy, can be converted into acetylcholine within the body. Acetylcholine plays a crucial role in various physiological processes, including muscle contraction and neurotransmission. By increasing the availability of choline, we aim to enhance the production of acetylcholine, potentially leading to improved muscle tone and skin firmness. This method could offer a novel approach to addressing issues related to skin aging and muscle strength, and further studies are needed to fully explore its efficacy and benefits. The outcome of the present investigation suggests that SKIN Colin^®^ is safe and well-tolerated and is effective in the treatment of skin damage. Skin Colin^®^ can significantly improve SASSQ assessment and GAIS with high safety and low incidence of side effects, which is worthy of clinical promotion probability also through an effect on the endothelial cells [35]. SKIN Colin^®^ is a relatively new skincare ingredient. Clinical data on its efficacy and safety in improving the appearance of aging facial skin shows that the use of SKIN Colin^®^ for the face via mesotherapy can safely improve skin firmness and reduce wrinkles. However, the results of the present study do not provide direct evidence that the administration via mesotherapy of acetylcholine to neuromuscular synaptic junctions is a key factor in enhancing facial muscle tensile strength and improving skin tone. Another method to evaluate the effectiveness of mesotherapy treatments is high-frequency ultrasound (HFUS), which can assess dermal changes, including thickness and echogenicity, providing quantitative data on skin rejuvenation [36]. In conclusion our results suggest that a course of treatment with choline via mesotherapy triggers a cascade of dynamic events that result in an improvement of SASSQ. This data is very important for possible fields of application in the treatment of skin and muscle aging. However, the present study has limitations due to the small sample size, the lack of a control group, and the fact that all participants were female. Therefore, it is necessary to conduct comparative studies that take gender into account to clarify these findings. Therefore, the results should be validated in a larger study.

## Figures and Tables

**Table 1:** Subject demography.

Male	N (%)	0 (0)
Female	N (%)	40 (100)
Age (years)	Median	48.0
	Range	38–67
BMI	Mean	26.5
	STD	2.4

N = number of subjects, STD = standard deviation.

**Table 2:** Subject satisfaction.

Satisfaction	Number (%) of Subjects
	Day 168
**Q1—Appearance after treatment (N = 40)**	
very much improved	15 (37.5)
much improved	17 (42.5)
slightly improved	8 (20.0)
no change	-
**Q2—Satisfaction with treatment (N = 40)**	
very satisfied	25 (62.5)
satisfied	15 (37.5)
not satisfied	-

**Table 3:** Adverse events by system organ class and preferred term (N = 40).

		All, N = 40	
	n	N′	(%)
Total	8	8	(20.0)
General dis. and ad. site cond.	8	8	(20.0)
Injection site redness	2	2	(5.0)
Injection site bruising	4	4	(10.0)
Injection site pain	2	2	(5.0)

n = number of events, N = number of subjects in data set, N′ = number of subjects with events, SAF = safety data set.

## Data Availability

Data can be obtained from the authors upon reasonable request.
